# Sex-dependent liver cancer xenograft models for predicting clinical data in the evaluation of anticancer drugs

**DOI:** 10.1186/s42826-021-00087-z

**Published:** 2021-02-25

**Authors:** Sungryong Oh, Joohee Jung

**Affiliations:** College of Pharmacy, Duksung Women’s University, #33, Samyang-ro 144-gil, Dobong-gu, Seoul, 01369 South Korea

**Keywords:** Doxorubicin, Sex difference, Cardiotoxicity, Liver cancer, Xenograft model, The Korea Institute of Drug Safety & risk management database

## Abstract

**Background:**

The incidence and mortality of liver cancer show a great difference between the sexes. We established sex-dependent liver cancer xenograft models and investigated whether such sex-dependent models could be used to simultaneously evaluate the therapeutic and adverse effects of anticancer drugs for drug screening.

**Results:**

In the in-vitro test, the cytotoxicity of anticancer drugs (cisplatin, 5-fluorouracil, and doxorubicin) was compared between male- and female-derived liver cancer cell lines. Cisplatin and 5-fluorouracil exhibited cytotoxicity without sex-difference, but doxorubicin showed dose-dependently significant cytotoxicity only in male-derived cells. Our results showed a strong correlation between preclinical and clinical data with the use of sex-dependent liver cancer xenograft models. Moreover, the male-derived Hep3B-derived xenograft model was more sensitive than the female-derived SNU-387-derived xenograft model against doxorubicin treatment. Doxorubicin showed more severe cardiotoxicity in the male xenograft model than in the female model. We investigated the occurrence frequency of doxorubicin-related cardiotoxicity using data obtained from the Korea Institute of Drug Safety & Risk Management Database, but no significant difference was observed between the sexes.

**Conclusions:**

Our results suggest that sex-dependent xenograft models are useful tools for evaluating the therapeutic and adverse effects of anticancer drugs, because sex is an important consideration in drug development.

**Supplementary Information:**

The online version contains supplementary material available at 10.1186/s42826-021-00087-z.

## Background

The incidence of liver cancer and the associated mortality considerably vary between the sexes [1]. Sex difference in incidence of cancer according to the causative chemical carcinogens have been reported [2]. Furthermore, the difference in mortality between males and females can be attributed to the difference in chemotherapeutic efficacy. Recent studies have suggested sex differences in the sensitivity to anticancer drug, due to adipocyte distribution, enzyme expression, and sex hormones [3–5]. However, previous preclinical studies of anticancer drugs have often neglected the sex of the experimental animals. Furthermore, male animals are mostly used in in-vivo studies [6]. This discrepancy between preclinical data and clinical data has been attributed to species difference. It has been reported that the sex difference between human and animal models influenced the outcome of studies in experimental rats after acute brain injury and those on autoimmune diseases using mice [7]. Similarly, it has been reported that liver cancer occurred more frequently in male animals than in females, as observed in humans [8, 9]. In the field of anticancer drug development, it is essential to establish a strong correlation between preclinical data and clinical data. Therefore, in the preclinical evaluation step, it is important to use experimental animal models in which the physiological conditions of humans can be extrapolated.

In liver cancer, doxorubicin (Dox), 5-fluorouracil (5-FU), and cisplatin (CDDP) have been classically used as chemotherapeutic agents [10]. These drugs inhibit the proliferation of cancer cells via various mechanisms: Dox generates free radicals and intercalates into DNA, 5-FU inhibits thymidylate synthase and interrupts the cell cycle as a uracil analogue, and CDDP induces DNA damage [10]. However, the action of these drugs is not specific to cancer cells. Thus, other normal tissues are also damaged depending on drug distribution, affinity, and accumulation. Dox, 5-FU, and CDDP are known to induce cardiotoxicity, neurotoxicity, and nephrotoxicity, respectively [11–13]. Several studies have focused on the cause of unexpected adverse effects and attempted to elucidate the underlying mechanism. For drug efficacy testing, various disease models (e.g., chemical-induced murine model, orthotopic xenograft model, ectopic xenograft model, and metastasis) have been utilized, whereas for toxicity testing, healthy animals have been used without considering the pathological conditions. Recently, sex has been considered one of these reasons for the pharmacokinetics and pharmacodynamics of these drugs. Various clinical data obtained from patients with cancer demonstrate sex differences. Thus, appropriate animal models should be used to predict drug efficacy and toxicity. In this study, we investigated whether sex-dependent evaluation models could be simultaneously used to evaluate the therapeutic and adverse effects of anticancer drugs in drug screening.

## Results

### Difference in cytotoxicity of anticancer drugs in male- and female-derived cell lines

**Fig. 1:**
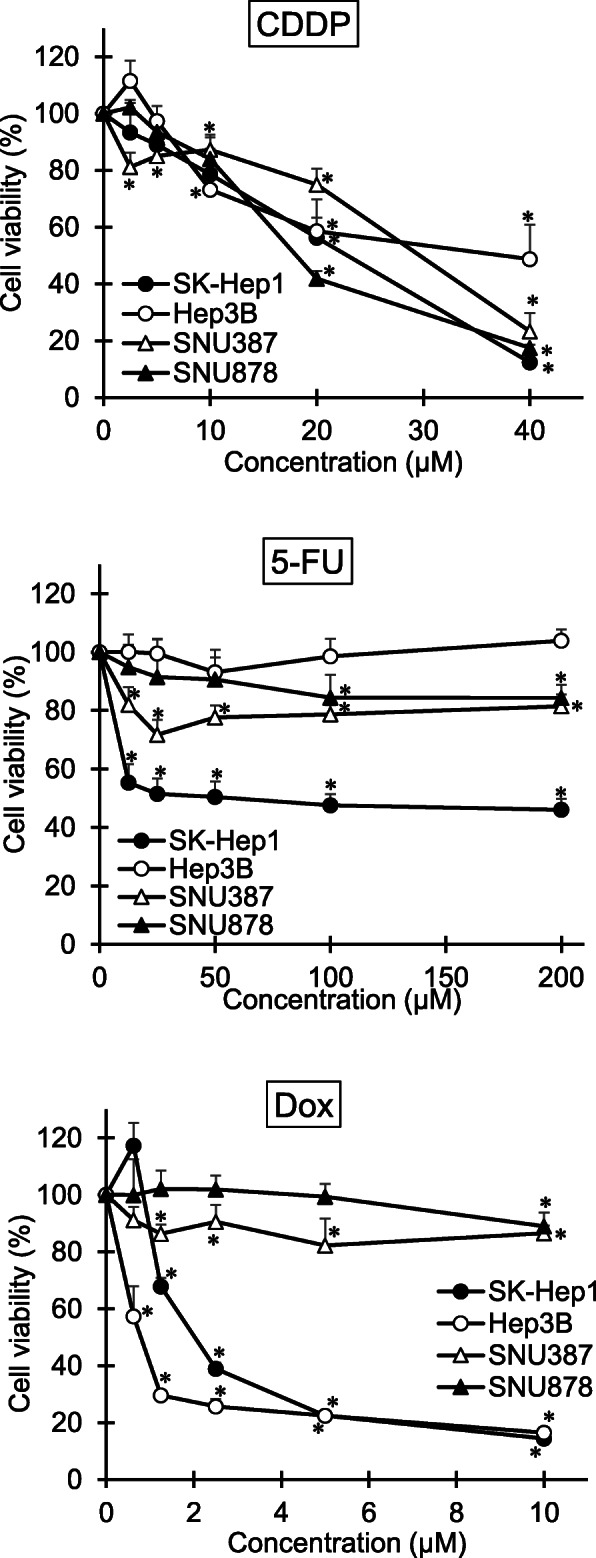
Comparison of anticancer drugs in liver cancer cell lines. Male-derived SK-Hep1 and Hep3B cells, and female-derived SNU-387 and SNU-878 cells were treated with Dox, 5-FU and CDDP for 24 h. Data are presented as mean ± standard deviation (*n* = 6). * *p* < 0.05 (one-way ANOVA test followed by Tukey’s post-hoc test)

The cytotoxicity of commercial anticancer drugs (Dox, 5-FU, and CDDP) was investigated in two male-derived and two female-derived liver cancer cell lines (Fig. 1). As shown in Fig. 1, CDDP substantially inhibited the viability of all liver cancer cell lines. 5-FU significantly inhibited the viability of cells as follows: SK-Hep1 > SNU387 > SNU878 cells. 5-FU concentrations up to 200 μM did not exert toxicity on Hep3B cells. Dox dose-dependently decreased the viability of Hep3B and SK-Hep1 cells, but rarely inhibited the growth of SNU387 and SNU878 cells. Interestingly, only male-originated cell lines were sensitive to Dox. Among the three tested anticancer drugs, Dox was selected as the target drug for further investigation to understand sex differences in the in-vivo models.

### Comparison of Dox sensitivity between the male- and female-derived liver cancer xenograft models

**Fig. 2:**
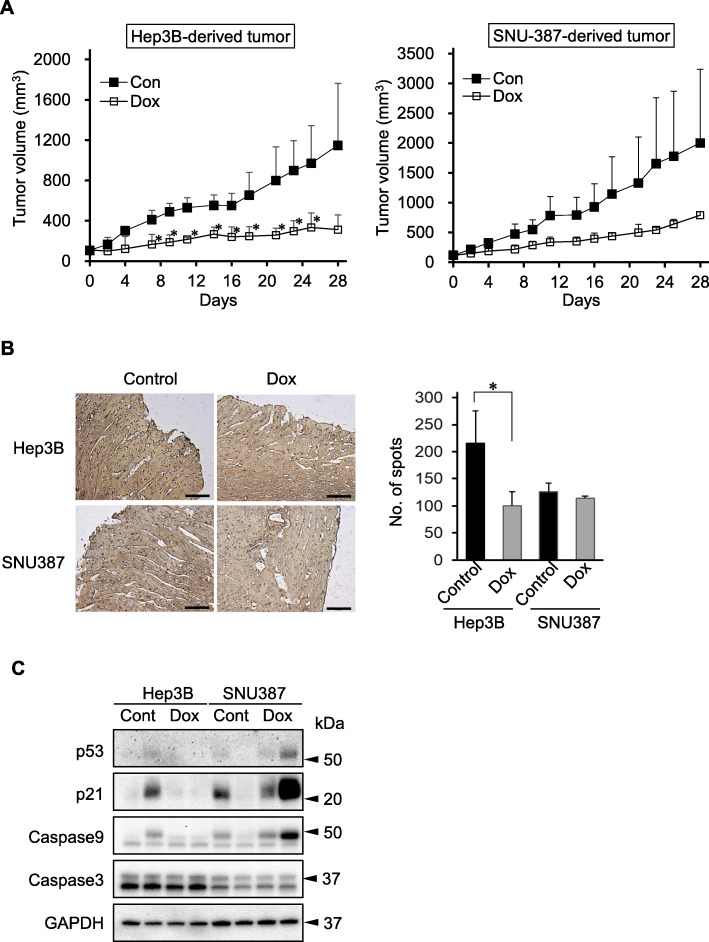
Difference of doxorubicin efficacy between male- and female-derived xenograft models. **a.** Tumor growth curve. Hep3B cells were transplanted in male Balb/c nu/nu mice and SNU-387 cells were in female mice. Data are presented as mean ± standard deviation (*n* = 4/group). * *p* < 0.05 (Student *t*-test). **b**. Expression of Ki-67 in the tumor tissues, determined by immunohistochemistry. *(Left panel)* Brown dots indicate Ki-67 expression. Scale bar, 100 μm (Right panel). Brown dots were counted using ImageJ. Data are shown as mean ± standard deviation (*n* = 3/group). * *p* < 0.05 (Student *t*-test). **c**. Apoptosis-related protein expression in tumor tissues, determined by western blotting. (Cont, control; Dox, doxorubicin)

Two types of xenograft models were prepared with male-derived Hep3B cells and female-derived SNU-387 cells. Except for the sex, the properties of the two cell lines are similar. These cell lines were derived from hepatocellular carcinomas infected with hepatitis B virus. Hep3B cells and SNU-387 cells were transplanted into male and female mice, respectively. As shown in Fig. 2a, Dox delayed tumor growth in both xenograft models (*p* < 0.05, three-way ANOVA test). A comparison of tumor growth between male and female mice revealed that the growth of Hep3B-derived tumor was significantly inhibited by Dox, whereas that of SNU-387-derived tumor was not. These results could be due to large individual deviations in the SNU-387-derived xenograft model. In the tumor tissues, the Dox-treated group showed lower expression of Ki-67 protein, a proliferation marker, than the control group (Fig. 2b). As shown in Fig. 2b (right panel), Dox significantly inhibited Ki-67 expression only in Hep3B-derived tumor tissue. These results suggest that Dox suppressed cell proliferation in Hep3B-derived tumor tissue. On the contrary, Dox induced the expression of p53 and p21, and increased the caspase 9 and cleaved caspase 9 levels only in SNU-387-derived tumors (Fig. 2c).

### Difference in Dox-induced toxicity between male- and female-derived liver cancer xenograft models

**Fig. 3:**
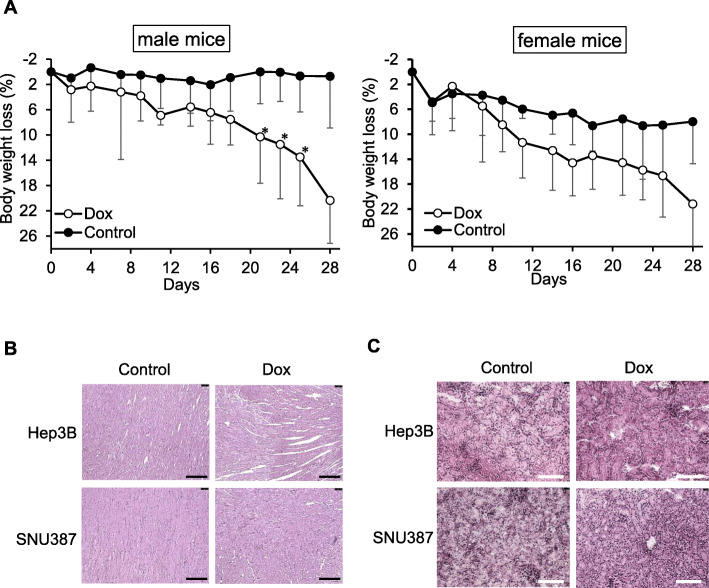
Difference between the adverse effect of doxorubicin in male- and female-derived xenograft models. **a**. Mouse’s body weight loss. Body weight of mice was measured during the detection of tumor growth. Data are presented as mean ± standard deviation (*n* = 4). * *p* < 0.05 (Student *t*-test). **b.** Histology of the left ventricle tissues. Scale bar, 200 μm. **c.** Histology of the kidney tissues. Scale bar, 100 μm. Tissue sections were stained with hematoxylin and observed under a microscope

To determine the efficacy of Dox in vitro and in vivo, we investigated whether the adverse effects of Dox were different between male- and female-derived xenograft models. Repeating treatment with Dox slightly induced a decrease in mouse body weight. In particular, the Hep3B-derived xenograft models were more sensitive than the SNU-387-derived xenograft models (Fig. 3a). As shown in Fig. 3b, Dox-induced cardiotoxicity was observed in the Hep3B-derived xenograft model, but not in the SNU-387-derived xenograft model. However, there was no difference in Dox-induced nephrotoxicity between the male- and female-derived xenograft models under the conditions of our study (Fig. 3c).

### Sex differences in Dox-induced cardiotoxicity investigated using data from the Korea Institute of Drug Safety & risk management (KIDS) database

Dox-induced cardiotoxicity in Korean clinical patients was investigated using data from the Korea Adverse Event Reporting System database from KIDS (KIDS-KAERS database) from 2012 to 2018. The number of adverse reactions induced by Dox was 7899. Adverse reactions of Dox were classified according to the World Health Organization-Adverse Reactions Terminology system organ classes (WHO-ART SOC) and preferred terms. Among them, there were 15 cases of cardiotoxicity, including cardiovascular disorders (WHO-ART SOC, 1010), myocardial, endocardial, pericardial, and valve disorders (WHO-ART SOC, 1020), and heart rate and rhythm disorders (WHO-ART SOC, 1030). Cardiotoxicity occurred in 6 female patients and 9 male patients (Table 1). Male patients prescribed medicine including Dox complained of cardiotoxicity more than female patients, but there was no significant difference.

**Table 1:** Dox-induced cardiotoxicity in KIDS-KAERS database

WHO-ART SOC	Case number ^1)^	
	Male	Female
Cardiovascular disorders (general) Heart rate and rhythm disorders Myocardial, endocardial, pericardial & valve disorders	9	6

^1)^ Adverse drug reaction was reported from 2012 to 2018 in KIDS-KAERS database

## Discussions

Sex differences in the occurrence of cancer and the associated mortality, as well as the therapeutic efficacy and adverse reactions of anticancer drugs have been studied and elucidated steadily. In the preclinical stage, there is demand for studies on sex difference in terms of efficacy and adverse reactions of anticancer drugs. Nevertheless, the application of sex as a bias factor is rare in in-vivo studies. In this study, we investigated whether sex-dependent xenograft models could be utilized to simultaneously evaluate the efficacy and adverse reactions of anticancer drugs in the preclinical stage. To select an anticancer drug for sex-dependent xenograft models, we compared the viability of four cell lines; based on the results, we selected Dox for further analysis. The susceptibility of cancer cells varies with drugs (Genomics of Drug Sensitivity in Cancer, https://www.cancerrxgene.org) [14]. Our results also showed differences in cell viability depending on anticancer drugs (Fig. 1). CDDP dose-dependently inhibited the viability of the four cell lines. 5-FU exerted cytotoxicity against three cell lines, except Hep3B cells. Fas-negative Hep3B cells have been reported to be resistant to 5-FU [15]. Dox substantially decreased the viability of SK-Hep1 and Hep3B cells. Dox significantly inhibited the viability of Hep3B and SK-Hep1 cells, but did not inhibit the growth of SNU387 and SNU878 cells. Interestingly, these results were found to be dependent on the sex of the cell lines. Further investigation is required to determine whether this result was due to the specificity or sex difference of cell lines. As Dox fit the aims of this study, it was used as the test drugs in the in-vivo study.

To prepare sex-dependent xenograft models, the sex of the donor cell lines was matched with that of the experimental animal. We observed differences in tumor growth between male and female mice, although the same cell line was transplanted into them (Supplementary data 1). In previous studies, we suggested that the sex of experimental animal should be considered to in in-vivo tests [16, 17]. Therefore, the therapeutic and adverse effects of Dox were compared using sex-dependent liver cancer xenograft models. A limitation of the study is that only the xenograft models that matched the sex of mouse and cell line were used in this study. Nevertheless, the results suggested that the Hep3B-derived xenograft model was more sensitive than the SNU-387-derived xenograft model against Dox treatment (Fig. 2a). Sex differences in the adverse effects of 5-FU have been reported in colorectal cancer models [16]. Our results and the findings of the previous study [16] indicate that sex is an important consideration in drug development. To elucidate these differences, we further investigated Ki-67 expression and apoptosis-related protein expression in tumor tissues (Fig. 2b and c). Dox significantly inhibited Ki-67 expression, indicating a delay in cell proliferation. Because Hep3B cells are p53-deficient [18], p53 and p21 (a target gene of p53) expression was not upregulated by Dox in Hep3B-derived tumor tissues. On the contrary, apoptosis-related protein levels were increased by Dox in SUN-387-derived tumor tissues. These results suggest that the specificity of transplanted cells is maintained in xenograft models.

In this study, we investigated the adverse effects of Dox in these xenograft models. Dox is known to induce cardiotoxicity [19] and nephrotoxicity [20]. Various studies have elucidated the mechanism of Dox-induced cardiotoxicity in rats [21, 22] and mice [23]. However, these studies focused on dose, treatment duration, and injection method of Dox to induce cardiotoxicity in healthy animals. Sex-dependent xenograft models might be used to mimic the reaction of Dox in patients with cancer. Our results showed that male xenograft models are more sensitive to Dox-induced cardiotoxicity and body weight loss than female models (Fig. 3). According to the data of the KIDS-KAERS database, cardiotoxicity is slightly higher in Korean men than in Korean women receiving the medication including Dox. The following information was not available in the KIDS-KAERS database: effects of treatment with combinations including Dox and Dox only, and the underlying conditions of patients. Thus, the result showed a trend of Dox-related cardiotoxicity, although no significant difference was observed between the sexes.

## Conclusions

Understanding sex differences in drug sensitivity while maintaining the quality of life of patients during chemotherapy is important for effective cancer therapy. In this study, the use of sex-dependent liver cancer xenograft models simultaneously showed the therapeutic efficacy and cardiotoxicity of Dox, and sex difference was compared in terms of the occurrence of Dox-related cardiotoxicity using data from the Korean adverse reporting database. Our results suggest that sex-dependent xenograft models are useful tools for evaluating the therapeutic and adverse effects of anticancer drugs. Therefore, a sex-dependent xenograft model is proposed as a screening model for anticancer drugs.

## Methods

### Cell culture

Human male liver cancer cells (SK-Hep1 and Hep3B cells) and human female liver cancer cells (SNU-387 and SNU-878 cells) from Korean Cell Line Bank (Seoul, Korea) were used in this study. SK-Hep1 cells in MEM medium (GenDEPOT, TX, USA), Hep3B cells in DMEM medium (GenDEPOT), and SNU-387 and SNU878 cells in RPMI-1640 medium (GenDEPOT) supplemented with 10% fetal bovine serum (YoungIn Frontiers, Seoul, Korea) and 1% penicillin-streptomycin solution (GenDEPOT) were maintained under 5% CO_2_ humidified atmosphere at 37 °C.

### Cytotoxicity test

Cells (5 × 10^3^ cells/well) were seeded in 96-well plates and incubated for 24 h. DOX, 5-FU, and CDDP (Sigma-Aldrich, MO, USA) were added to the cells for 24 h. Subsequently, 3-(4,5-dimethylthiazol-2-yl)-2,5-diphenyltetrazolium (MTT, final concentration 0.5 mg/mL, Sigma-Aldrich) was added to each well, and then the cells were incubated at 37 °C for 3 h, and the supernatant was gently removed and discarded. One hundred microliter of dimethyl sulfoxide (Sigma-Aldrich) was added to each well at 37 °C for 30 min, and the absorbance (560 nm) of the solution was determined using a microplate reader (Infinite M200 PRO; Tecan Inc., Grödig, Austria) [24]. The data are shown as mean ± standard deviation (*n* = 6).

### Experimental animals

The study was performed in accordance with the guidelines for the care and use of laboratory animals and approved by the Institutional Animal Care and Use Committee of Duksung Women’s University (2018–003-001). Five-week-old female and male BALB/c nude mice were supplied by JUNGAH BIO (Gyeonggi, Korea). Animals were acclimatized for 1 week in the animal laboratory room. The conditions were as follows: 28 °C, 50% humidity, and 12/12-h light/dark cycle. Feed and drinking water were provided ad libitum.

### Experimental design

This study was designed to evaluate the sex difference of liver cancer xenograft models in response to Dox treatment. A comparison of the effect of Dox between male- and female-derived liver cancer xenograft models was investigated. The effect of Dox on liver cancer and other organs was evaluated. To prepare xenograft models, Hep3B cells (3 × 10^6^ cells/mouse) and SNU-387 cells (1 × 10^7^ cells/mouse) were subcutaneously injected into male and female BALB/c nude mice, respectively. When tumor volumes reached approximately 100 mm^3^, the mice were randomly divided into two groups (control group and Dox-treated group). Dox (5 mg/kg) was intravenously injected into mice three times per week for 4 weeks [25]. Tumor size and body weight were measured three times per week. Tumor volume was calculated as 0.5 × the longest length × (the shortest length)^2^ [17]. After the final detection, the mice were sacrificed, and tumor tissues and other organs were isolated. To compare the effect of Dox between female- and male-derived liver cancer xenograft mice, histological analysis, western blotting analysis, and immunohistochemistry were conducted.

### Tissue sections

The left ventricle was fixed in 4% para-formaldehyde solution, dehydrated, and embedded in paraffin wax. Paraffin blocks were cut into 5 μm sections using a microtome (Leica, Nussloch, Germany). The tumor tissues and kidneys were embedded in optical cutting temperature compound (Leica). Mold containing the tissue block was placed in liquid nitrogen and frozen completely. The frozen tissue blocks were sliced to 5 μm sections at − 20 °C using cryostat (Leica).

### Hematoxylin staining

For hematoxylin staining, paraffin tissue sections were warmed at 55 °C in an oven, deparaffinized in xylene three times 5 min each, and rehydrated in an ethanol series (100 to 50%) for 3 min each. The tissue sections were stained with hematoxylin solution (Sigma-Aldrich) and washed in water. For frozen tissue sections, they were fixed in alcohol, and then stained with hematoxylin solution. The tissue sections were covered by mounting solution (Sigma-Aldrich) and observed using a microscope (Leica).

### Immunohistochemistry

Frozen tumor tissue sections were washed in PBS, incubated in 0.3% H_2_O_2_ solution for 10 min, and rinsed in PBS. They were blocked with 5% albumin (GenDEPOT) for 1 h and incubated with anti-Ki-67 antibody (1:100; ab16667, Abcam, Cambridge, UK) overnight at 4 °C as previously described [26]. The rinsed tissue slides were incubated with the secondary antibody for 1 h at room temperature. The expression of Ki-67 was visualized using a DAB peroxidase substrate kit (Vector Laboratories Inc., CA, USA). The tissue sections were washed in water, covered with mounting solution, and observed using a microscope (Leica).

### Western blotting

Tumor tissues were homogenized in a radioimmunoprecipitation assay (RIPA) buffer (GenDEPOT) containing a protease and phosphatase inhibitor cocktail (GenDEPOT) as previously described [26]. Proteins were separated by sodium dodecyl sulphate-polyacrylamide gel electrophoresis and transferred on to polyvinylidene fluoride membranes (0.45 μm pore size; Millipore, Darmstadt, Germany). The membranes were blocked with 5% skimmed milk in Tris-buffered saline supplemented with 0.1% Tween 20 and incubated with primary antibodies against p53 (1:1000; Merck KGaA, Darmstadt, Germany), p21WAF1/Cip1 (1:2000; Merck KGaA), caspase 9 (1:1000; Cell Signaling Technology, Inc., MA, USA), caspase 3 (1:1000; Cell signaling technology, Inc.), and GAPDH (1:5000, Enzo Life Science, Inc., NY, USA) at 4 °C overnight. After incubating with the secondary antibody (1:3000; BioRad Laboratories, Inc., CA, USA) at room temperature for 3 h and washing, the blots were visualized using enhanced chemiluminescence solution and observed using ChemiDox™ imager (FluorChemE, Wiesbaden, Germany).

### Analysis of KIDS-KAERS database

Data of reported adverse reactions from 2012 to 2018 were obtained from the KIDS-KAERS database, an adverse event reporting system database. In KIDS-KAERS database, Dox-induced adverse reactions were selected. Among them, cases of heart-related disorders from WHO-ART SOC guidance and sexes were classified.

### Statistical analysis

Differences in data were considered statistically significant at *p* < 0.05, using Student *t*-test, one-way ANOVA followed by Tukey’s post-hoc test, and three-way ANOVA (GraphPad Prism 9; GraphPad Software Inc., CA, USA).

## Supplementary Information

**Additional file 1: Supplementary data 1**. Sex difference in SK-Hep1-derived tumor growth. SK-Hep1 cells (1 × 10^6^ cells/mouse) were subcutaneously injected into male and female BALB/c nude mice. Data are represented as the mean ± standard deviation (*n* = 5). * *p* < 0.05 (t-test).

## Data Availability

The dataset supporting the conclusions of this article are included within the article.

## Abbreviations

Dox: Doxorubicin
5-FU: 5-fluorouracil
CDDP: Cisplatin
RIPA buffer: Radioimmunoprecipitation assay buffer
KIDS-KAERS database: The Korea Adverse Event Reporting System database from the Korea Institute of Drug Safety and Risk Management
WHO-ART SOC: World Health Organization-Adverse Reactions Terminology system organ classes
