# Supplementary material for: Sex-dependent liver cancer xenograft models for predicting clinical data in the evaluation of anticancer drugs
Source: Lab Anim Res. 2021 Feb 25;37:10. doi: 10.1186/s42826-021-00087-z (PMC7905914; doi:10.1186/s42826-021-00087-z)
Supplement: Supplementary file 1 — Additional file 1: Supplementary data 1. Sex difference in SK-Hep1-derived tumor growth. SK-Hep1 cells (1 × 10^6^ cells/mouse) were subcutaneously injected into male and female BALB/c nude mice. Data are represented as the mean ± standard deviation (n = 5). * p < 0.05 (t-test). [file 42826_2021_87_MOESM1_ESM.pptx]

## Slide 1
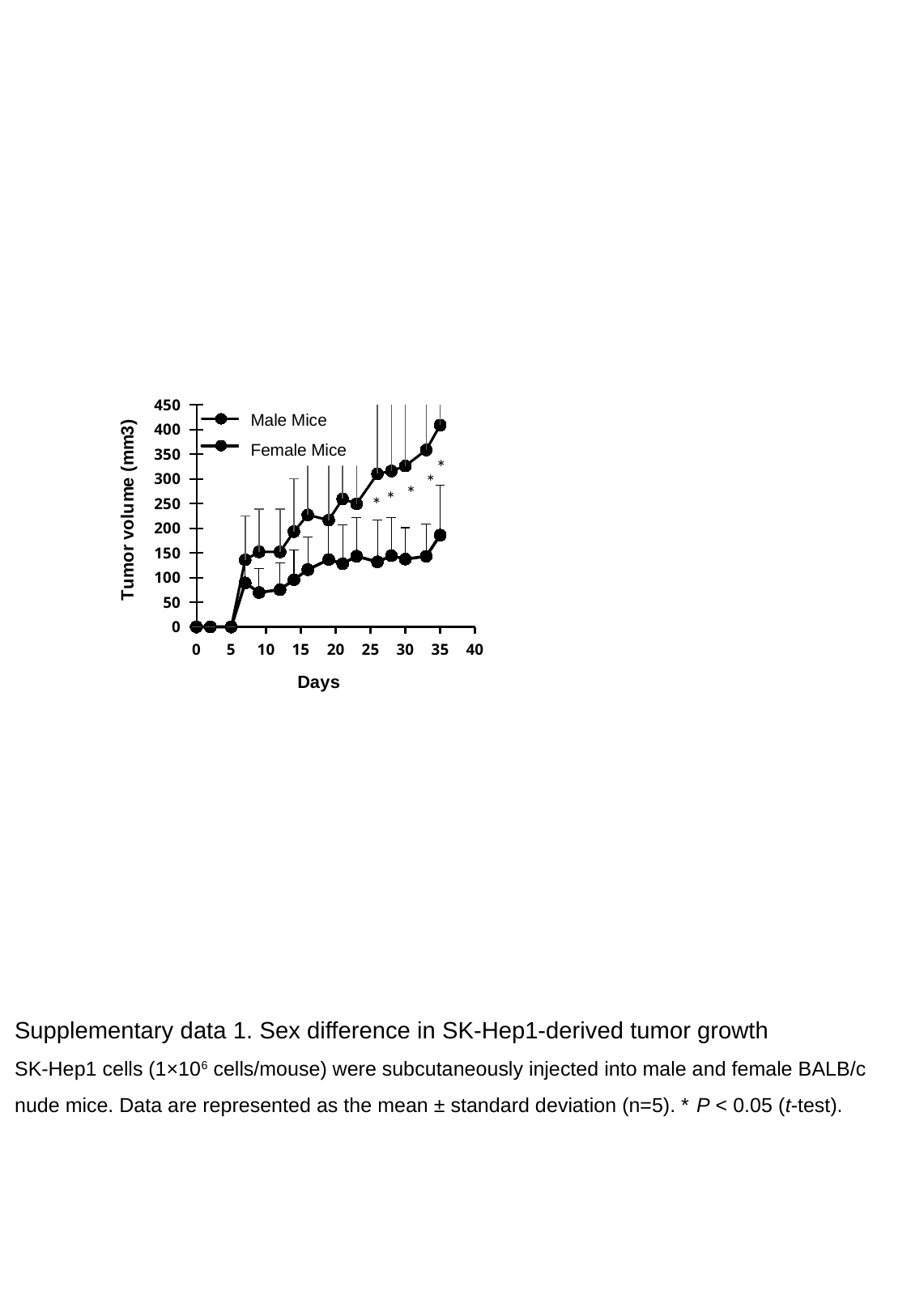

### Chart
| Category | Male | Female |
|---|---|---|Male Mice
Female Mice
*
*
*
*
*
Supplementary data 1. Sex difference in SK-Hep1-derived tumor growth
SK-Hep1 cells (1×10^6^ cells/mouse) were subcutaneously injected into male and female BALB/c nude mice. Data are represented as the mean ± standard deviation (n=5). * P < 0.05 (t-test).
